# Effective harvesting of microalgae by coagulation–flotation

**DOI:** 10.1098/rsos.170867

**Published:** 2017-11-15

**Authors:** Ling Xia, Yinta Li, Rong Huang, Shaoxian Song

**Affiliations:** 1 Hubei Key Laboratory of Mineral Resources Processing and Environment, Wuhan University of Technology, Luoshi Road 122, Wuhan, Hubei 430070, People's Republic of China; 2 School of Resources and Environmental Engineering, Wuhan University of Technology, Luoshi Road 122, Wuhan, Hubei 430070, People's Republic of China; 3 Doctorado Institucional de Ingeniería y Ciencia de Materiales, Universidad Autonoma de San Luis Potosi, Av. Sierra Leona 530, San Luis Potosi, C.P. 78210, Mexico

**Keywords:** microalgae, coagulation–flotation, dissolved organic matter, hydrophobicity, harvest, lipid

## Abstract

This study developed a coagulation–flotation process for microalgae *Chlorella* sp. XJ-445 harvesting, which was composed of algal surface modification by combined use of Al^3+^ and cetyltrimethylammonium bromide (CTAB) and followed dispersed bubble flotation. Dissolved organic matter (DOM) in the medium was firstly characterized and mainly consisted of hydrophilic low molecular weight molecules. The dosage of collector (CTAB) and coagulant (Al^3+^) were optimized, and with the pretreatment of 40 mg Al^3+^ and 60 mg CTAB per 1 g dry biomass without pH adjustment, a maximum flotation recovery efficiency of 98.73% can be achieved with the presence of DOM. Algal cells characterization results showed that the combined use of CTAB and Al^3+^ largely enhanced the algal floc size, and exhibited higher degree of hydrophobicity, which favoured the flotation, and can be interpreted by DLVO (Derjaguin, Landau, Verwey and Overbeek) modelling. A benefit in fatty acid conversion was further found with the optimized coagulation–flotation process. It was suggested that this coagulation based flotation is a promising strategy for high-efficiency harvesting of microalgae.

## 1. Introduction

Microalgae as an alternative feedstock for biodiesel production has attracted much attention due to their high lipid content, high photosynthetic efficiency, CO_2_ reduction in the environment, and producing different value-added products compared to conventional crops. However, commercial microalgae-based biofuel production is not economically viable yet. Discounting the not inconsiderable costs and difficulties associated with biomass production, the process of harvesting and dewatering has often been cited as one of the major factors preventing a scalable industry [1]. This is because the algal cells are small (commonly under 30 µm) and fragile, with a density similar to that of water which makes settlement and flocculation problematic. As reported, the algal biomass harvesting represents 20–30% of the total biomass production costs [2].

Typically, the main methods that are researched for microalgal biomass separation are centrifugation, filtration, coagulation/flocculation, gravity sedimentation, flotation or electrical approaches. Centrifugal recovery is the fastest and most reliable method for biomass recovery for a wide range of species, however, the most expensive due to its high energy consumption (about 3000 kWh t^−1^) [3]. Filtration is common, but only sustainable for harvesting microalgae of long length or those forming large colonies [4], and it also suffers from the problem of membrane contamination. Gravity sedimentation is more cell size dependent and is generally used for sewage-cultured algae recovery; however, it is time-consuming and needs further thickening procedure with high moisture [2]. Electrocoagulation and electroflotation seem effective but are rarely used in harvesting microalgae [5]. Coagulation is considered a promising harvesting method of low cost for bulk biomass production [6]; however, it is usually followed by sedimentation, filtration or flotation to concentrate the biomass. Among these approaches, flotation is known as a more effective and economic way to harvest microalgae by taking advantage of their natural characteristics of relatively low density and self-float [7]. For flotation success, generally, the bubbles are negatively charged with large size and hydrophobic (HPO) surface. Thus, a lot of efforts have been made to create bubbles of smaller size for collecting algal cells [8,9] instead of increasing the size of algal particles using the normal size of bubble to cut the energy input. Moreover, there is also a missing link between flotation performance and algal surface properties in terms of the degree of hydrophobicity and algal floc size [10,11], which are key factors for flotation succeeding. The most used and effective methods for forming algal flocs to enhance the collision activity of algal cells with bubbles is to add chemicals, such as aluminium salts, into the algal suspensions. The flocs provide greater bubble formation at the surface, bubble entrapment and bubble entrainment due to the larger surface area, which would favour the algae removal. In addition, when using the flotation, an addition of cationic surfactant, e.g. cetyl trimethylammonium bromide (CTAB) can generate bubbles, improve the electrostatic interactions between the bubbles and the algal cells, and eventually benefit the algae removal [12]. Thus, the combination of coagulant and the surfactant may serve as a better strategy for algal flotation. This study is emphasized on designing a process of coagulation-based flotation by firstly modifying the properties of algal cells by adding Al^3+^ and CTAB for oleaginous algal biomass recovery.

The aim of this study is to evaluate the harvesting potential of the modified chemical addition using a simple foam flotation device. Initial optimization trials of flotation were conducted using different concentrations of collector (CTAB) and coagulant (Al^3+^) to modify the surface properties of algal cells for better flotation with the existence of dissolved organic matter (DOM). The surface characterization and fatty acid profiles of algal cells were monitored to evaluate the coagulation–flotation process and its influence on the final biodiesel production. The coagulation process was also interpreted by DLVO (Derjaguin, Landau, Verwey and Overbeek) theory in this study.

## 2. Material and methods

### 2.1. Organism and culture

The organism *Chlorella* sp. XJ-445 was provided by prof. Hu Chunxiang from Institute of Hydrobiology, Chinese Academy of Sciences, China. It was isolated from northern Xinjiang province of China and identified by classical morphological methods. Stock cultures were grown in BG-11 medium containing 1.5 g NaNO_3_, 30 mg K_2_HPO_4_, 36 mg CaCl_2_ · 2H_2_O, 6 mg ammonium citrate monohydrate, 6 mg ammonium ferric citrate, 1 mg EDTA, 2.86 µg H_3_BO_3_, 1.81 µg MnCl_2_ · 4H_2_O, 0.222 µg ZnSO_4_ · 7H_2_O, 0.39 µg NaMoO_4_ · 5H_2_O, 0.079 µg CuSO_4_ · 5H_2_O, 0.050 µg CoCl_2_ · 6H_2_O in 1 l sterile distilled water [13].

### 2.2. Dispersed air flotation test

Flotation experiments were carried out using a columnar 150 ml flotation cell (45 mm in diameter and 100 mm in height). There is a porous gas diffusor with a nominal pore diameter placing at the column bottom, just above the gas inlet port. Dispersed air bubbles (approx. 700 µm) were generated from an air blower through the diffusor. The gas pressure leaving the blower was approximately 0.01 MPa and the flow rate to the column was 50 ml min^−1^ controlled by a flowmeter. The bubbles picked up microalgal cells and rose to the top without stirring, forming a microalgae-laden froth, which was subsequently collected manually. Prior to the flotation process, the algal cultures were well mixed and subdivided into aliquots of 100 ml at a concentration of 0.5 g l^−1^, and transferred into the flotation cell. The batch flotation harvesting tests were conducted in triplicate with a batch run time of 15 min. For combined use of CTAB and Al^3+^ (as a form of aluminium sulphate), algal cells were firstly coagulated with different concentrations of Al^3+^, and then CTAB was added into the flocs at a given concentration and followed by pH adjustment using 0.1 M NaOH or HCl.

Harvest efficiency (Y) based on optical density measurements were determined to evaluate flotation performances and calculated according to the following equation (2.1) [14]:

2.1 $$Y(\%)=\left(1-\frac{\text{O}{\text{D}}_{a}V_{a}}{\text{O}{\text{D}}_{i}V_{i}}\right)\times 100\%,$$

where OD_i_ and *V*_i_ are the initial optical density at 680 nm and the volume of algal suspension (before chemical addition and flotation). OD_a_ and *V*_a_ are, respectively, the optical density at 680 nm of the aqueous phase, the volume of the aqueous phase after flotation process.

### 2.3. Analytical methods

#### 2.3.1. Dissolved organic matter characterization

After 7 days cultivation, when the algal cells reached a biomass concentration of about 1.0 g l^−1^, they were centrifugally harvested at 6000g, and the supernatant was collected for DOM analysis. The determination of hydrophilic (HPI) and hydrophobic fractions in DOM was performed following the method described by Malcolm and MacCarthy [15]. Briefly, DOM samples of 250 ml with pH adjusted to 2 (with 1 M HCl) were passed consecutively through the serially connected two 15 ml columns, which were filled with 50 ml of XAD-8 and XAD-4 resin (Amberlite, USA), respectively. The hydrophobic fraction was adsorbed on XAD-8 resin, the transphilic (TPI) fraction on XAD-4 resin and non-retained compounds represented HPI fraction. Adsorbed HPO and TPI fractions were eluted from the resins with 150 ml of NaOH (0.1 M). Flow rates of filtration and elution were both controlled at 1 ml min^−1^. Concentrations of each fraction were determined by dissolved organic carbon (DOC) measurements (TOC-V_CPH_, Shimadzu).

The total DOM and DOM fractions were characterized in terms of molecular weight (MW) distribution. Centrifugation (4000g, 30 min) at 4°C was used to drive the DOM fraction through Amicon Ultra-15 centrifugal filters (Millipore, USA) of 100, 50, 30, 10, 3 kDa. The MW distribution was also expressed as DOC.

#### 2.3.2. Characterization of algal cells

The laser diffraction technique was widely used in the measurement of algal floc size [16,17]. It was also employed in this study using a Malvern HYDRO 2000 MU Laser particle size analyser (UK). The surface charge of algal cells/flocs was measured by a zeta potential analyser (Malvern, Zetasizer Nano, ZS90).

According to the previous studies, contact angles can better explain the hydrophobicity of the algal cells surface, thus contact angles were measured using the sessile drop technique with water as the reference liquid in this study [18]. Briefly, the harvested cells with and without dosage addition were filtered on 0.45 μm mixed cellulose acetate filter with thickness of approximately 250 µm. The lawns were then placed in a Petri dish on the top of 1% (weight/volume) agar layer containing 10% glycerol (v/v) and stored for 2 h to homogenize the moisture content. The filters were then dried in air for 60 min to obtain relatively stable contact angles. Contact angles were determined using a tensiometer (K100, Krüss GmbH, Hamburg, Germany).

#### 2.3.3 DLVO modelling

The DLVO theory (Derjaguin, Landau, Verwey and Overbeek theory) was firstly developed to relate the stability of colloidal suspensions to the total potential energy between two particles. In the theory, the net interaction energy between particles is interpreted as a balance of attractive Van der Waals force (*G*_LW_) and an electrostatic force (*G*_EL_), which is usually repulsive because of overlapping electrical double layers surrounding charged particles. Thus, the total energy of interaction (*G*_TOT_) between two particles (microalgal cells in this study) can be obtained by the summation of interaction energy, resulting from *G*_LW_ and the overlap of electrical double layers associated with the charged surfaces *G*_EL_. Both *G*_LW_ and *G*_EL_ are interpreted as functions of the separation distance (*H*) between cells [19]. Equations (2.2)–(2.4) governing the interactions are as follows [20]:

2.2 $$\begin{array}{r} G_{\mathrm{TOT}}(H)=G_{\mathrm{LW}}(H)+G_{\mathrm{EL}}(H), \end{array}$$

2.3 $$\begin{array}{rl} G_{\mathrm{LW}}(H) & =-\frac{AR}{12H} \end{array}$$

2.4 $$\begin{array}{r} \;\text{and}\;G_{\mathrm{EL}}(H)=2\pi \varepsilon R\varphi^{2}\text{In}[1+\exp(-kH)], \end{array}$$

where *A* is the Hamaker constant, which is a strain-dependent parameter. Here, the value is 8.1 × 10^−21^ J for *Chlorella* sp. XJ-445 [21]. *R* is the cell radius (approx. 3.57 µm) [21]. *ε* is the dielectric constant of the solution (80 × 8.854 × 10^−12^ C^2^ J^−1^ m^−1^ for aqueous). *φ* and *k* stand for the cell surface zeta potential and the reciprocal of the Debye length, respectively. *k* can be obtained by the following equation (2.5) [22]:

2.5 $$k=3.28\times 10^{9}\times I^{0.5},$$

where *I* is the ionic strength in terms of molarity.

In the classic DLVO theory, the Van der Waals force is responsible for attraction, thus, the corresponding interaction energy, *G*_LW_, is generally negative while the interaction energy resulting from the electrostatic repulsion force, *G*_EL_, is positive. According to equations (2.3) and (2.4), *G*_LW_ decreases with the increasing separation distance between the cells, and *G*_EL_ is an approximately exponential function of the separation distance with a range of the order of the thickness of the double layer (*κ*^−1^). Consequently, *G*_LW_ predominates at small intercellular distance and *G*_EL_ dominates at intermediate separation distance between cells. Hence, at larger energy barrier, the cell suspension is more stable, and the flocculation is prevented. According to the DLVO model, only the electrostatic double layer force can be significantly modified, and repulsion performance between cells can be greatly affected by changing the ionic strength of the liquors medium or by modifying the surface charge of the cells through pH adjustment or addition of positively charged flocculants [20,22]. Thus, it was employed in this study to interpret the algal coagulation process in this study.

#### 2.3.4. Lipid analysis

Total lipid content of the cells was determined in triplicate by the modified Bligh & Dyer [23] method using 1 : 2 chloroform : methanol and presented as weight percentage (%) [23]. Fatty acids were determined as fatty acid methyl esters (FAMEs) in triplicate after acidic transesterification of lipids [24]. The resulting FAMEs were analysed by gas chromatograph mass spectrometry (GC-MS; Thermo Scientific ITQ 700, USA) equipped with a flame ionization detector (FID) and a fused silica capillary column (60 m × 0.25 mm × 0.25 µm; Agilent Technologies, USA). The injector and detector temperatures were maintained at 270 and 280°C, respectively, with an oven temperature gradient of 50–170°C at 40°C min^−1^ after a 1 min hold time at 50°C, then with an oven temperature gradient of 170–210°C at 18°C min^−1^ after a 1 min hold. All parameters of the FAME were derived from the calibration curves generated from the FAME standard mix (Supelco 37 component FAME mix, Sigma-Aldrich, USA) [25].

### 2.4. Statistical analysis

In this study, *t-*test was performed using SPSS 18.0 package (SPSS, Chicago, IL, USA), with values of 0.05 selected as significance.

## 3. Results and discussion

### 3.1. Characterization of dissolved organic matter

**Figure 1.**
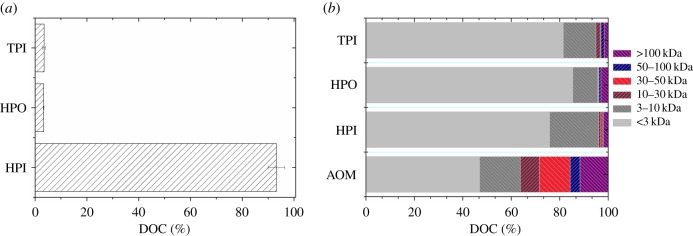
Hydrophobic, hydrophilic and transphilic fraction of DOM (*a*) and the relative molecular weight fractionations of DOM and its fractions (*b*).

Figure 1*a* shows the hydrophilic (HPI) and hydrophobic (HPO) properties of DOM of *Chlorella* sp. XJ-445 at exponential phases. In terms of DOC, HPI fractions dominated the DOM, constituting above 90% of DOM, whereas HPO and transphilic (TPI) only accounted for 3.3% and 3.5% of DOM, respectively. This observation on high proportion of hydrophilic property of DOM was consistent with the results reported previously by other researchers [26,27].

Figure 1*b* illustrates the relative molecular weight fractionations of DOM and its HPI, HPO and TPI fractions obtained by centrifugation-driven filtration in terms of DOC. The largest portion of compounds was determined in less than 3 kDa fraction for DOM, followed by 3–10 kDa and greater than 100 kDa. For DOM, less than 3 kDa fraction accounted for almost 50% of algogenic organic matter (AOM), while over 70% for its three parts. This fraction represents low-MW intermediate products of metabolism such as aldehydes, hydrocarbons, amino acids as well as mono- and oligosaccharides [26]. Molecules with MW of 3–10 kDa, representing the similar character to those in 0–3 kDa, were the second largest fraction, which can be assigned to the metabolic products, for example, extracellular enzymes or their components [26]. The third largest fraction larger than 100 kDa probably includes high-MW and storage polysaccharides as well as starch in the form of amylose and amylopectin which probably originated from the dead body of green algal cells. Thus, the DOM of XJ-445 mainly consisted of hydrophilic low-MW molecules, which would inevitably increase the dosing reagents [28,29]. The following harvesting experiments were all using the algal culture with the presence of DOM, which presented a real harvesting condition for further application. Before flotation tests, a coagulating process was a necessity not only for the small-sized algal cells but also for the hydrophilic small-sized DOM.

### 3.2. Algal harvest by coagulation–flotation

#### 3.2.1. Changing collector dosage

**Figure 2.**
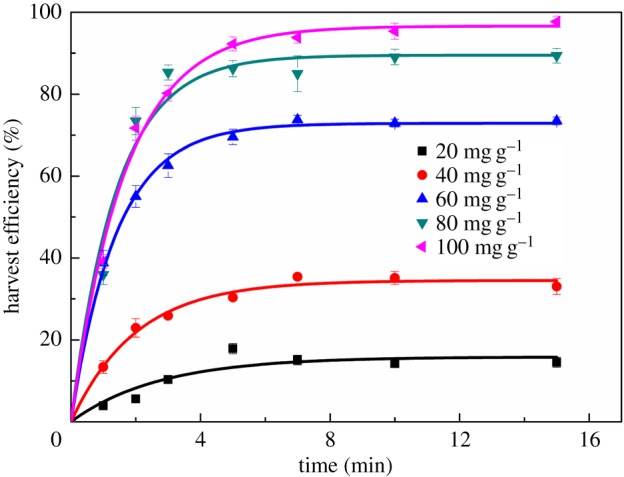
Kinetics of flotation of *Chlorella* sp. XJ-445 under different CTAB concentrations. The experimental data were fitted to equation (3.1).

Most microalgae are negatively charged at nature pH values and show a characteristic of low hydrophobicity, let alone with the hydrophilic DOM in the medium. Thus, to increase the degree of hydrophobicity, the microalgal cells were firstly pretreated using cationic collector tetradecyl trimethylammonium bromide (C_14_TAB) to evaluate the feasibility of harvesting *Chlorella* sp. XJ-445 using flotation. The flotation recovery efficiency of microalgae *Chlorella* sp. XJ-445 as a function of time under different CTAB concentrations was presented in figure 2. As shown, at a given flotation time, increasing the dosage of CTAB obviously increased the flotation efficiency, and an accomplishment of almost 90% of algal cells were recovered within 5 min. The observation was consistent with previous study that the higher the collector dosage, the higher removal efficiency of algal cells achieved [30].

The experimental was then fitted to the first-order chemical reaction analogy as following equation (3.1) [11].

3.1 $$Y=Y_{\max}(1-{\text{e}}^{-bt}),$$

where *Y*_max_ is the maximum flotation efficiency when the flotation time *t* approaches infinity, and *b* is the flotation rate constant.

The fitting parameters were listed in table 1. It showed that the experimental data were well fitted by equation (3.1) with the coefficients of determination (*R*^2^) all above 0.9, suggesting that the flotation process can be satisfactorily modelled by equation (3.1). In addition, the value of *b* increased with the increasing collector dosage from 20 to 60 mg g^−1^, however, decreased when further increasing the dosage. It was suggested that the highest flotation rate was found at a concentration of 60 mg g^−1^ CTAB as collector. Moreover, it was noted that when the concentration of CTAB was over 60 mg g^−1^ dry biomass, the harvest efficiency increased slowly with the increasing CTAB concentration. Considering the higher expense of CTAB, a concentration of 60 mg g^−1^ was chosen to combine a cheap coagulant for algal cells recovery in the subsequent experiments.

**Table 1.** Fitting parameters of flotation recovery data of *Chlorella* sp. XJ-445 under different CTAB concentrations using equation (3.1).

CTAB concentration (mg g^−1^ dry cell weight)	*Y*_max_ (%)	*b* (min^−1^)	*R*^2^
20	15.83	0.369	0.9079
40	34.50	0.500	0.9886
60	72.88	0.711	0.9975
80	89.51	0.710	0.9721
100	93.60	0.598	0.9942

#### 3.2.2. Changing coagulant dosage

**Figure 3.**
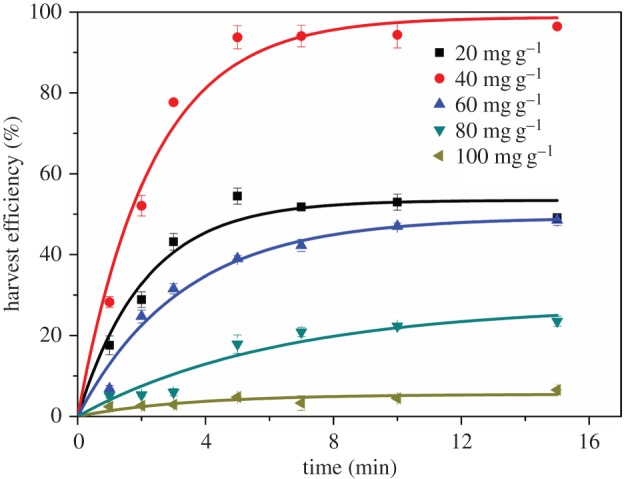
Kinetics of flotation of *Chlorella* sp. XJ-445 under different Al^3+^ concentrations with the presence of 60 mg g^−1^ CTAB. The experimental data were fitted to equation (3.1).

To further enhance the recovery efficiency of algal cells, a composite chemical combining coagulant (Al^3+^) and collector was introduced in this study. Aluminium salt was used because of its effective performance and reasonable price [28]. The coagulant was optimized using different concentrations of Al^3+^ to pretreat the cells and followed by adding 60 mg g^−1^ dry weight biomass CTAB as collector. As illustrated in figure 3, the harvest efficiency increased with increasing Al^3+^ concentration at a given time, peaked at 40 mg g^−1^ dry biomass Al^3+^, and then kept decreasing when further increasing Al^3+^ concentration. As fitted to equation (3.1), the highest maximum harvest efficiency achieved 98.73% (table 2), and it was approached by the experimental data of 96.43% at 15 min. Also, the maximum flotation efficiency was higher than that only using 100 mg g^−1^ dry biomass of CTAB, valued at 93.60%. Higher Al^3+^ concentration always achieved higher coagulation efficiency [31] even with the existence of DOM; however, there may be lower recovery efficiency using flotation [31]. It is worth mentioning that when adding Al^3+^ at a lower concentration of 20 mg g^−1^, the algae removal was inhibited compared to that only using 60 mg g^−1^ CTAB. It may be because that the increasing ion strength caused by aluminium sulphate caused the increase of gas bubbles [32], whereas the forming algal flocs were not large enough to react with them. This study also reported a tendency that higher Al^3+^ did not show significantly higher flotation recovery efficiency. Because higher ion strength not only led to larger gas bubbles but also led to a tendency of bubble rupture more easily [32], which would extensively inhibit bubble–cell adhesion [2]. Therefore, under the moderate Al^3+^ concentration (40 mg g^−1^), the interaction between air bubbles and algal flocs was optimized and the highest algal removal achieved.

**Table 2.** Fitting parameters of flotation recovery data of *Chlorella* sp. XJ-445 under different Al^3+^ concentrations with the presence of 60 mg g^−1^ CTAB using equation (3.1).

Al^3+^ concentration (mg g^−1^ dry cell weight)	*Y*_max_ (%)	*b* (min^−1^)	*R*^2^
20	54.42	0.286	0.7595
40	98.73	0.431	0.9806
60	53.43	0.475	0.9650
80	49.19	0.305	0.9761
100	27.53	0.159	0.9221

#### 3.2.3. Effect of pH

**Figure 4.**
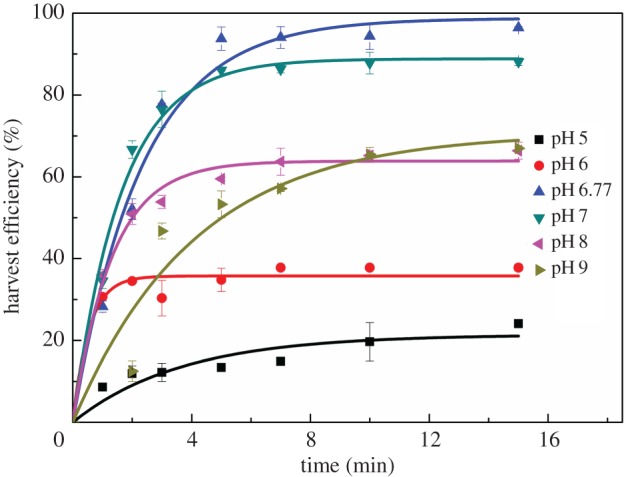
Kinetics of flotation of *Chlorella* sp. XJ-445 under different pH values with the presence of 40 mg Al^3+^ and 60 mg CTAB/g dry weight biomass. The experimental data were fitted to equation (3.1).

To investigate the effect of pH value on the recovery efficiency of algal flocs with the composite chemicals, the pH values of algal flocs were adjusted to the range of 5–9 before flotation. As illustrated in figure 4, the harvest efficiency increased with increasing pH from 6 to 6.77 (initial pH with composite CTAB and Al^3+^ treatment), and then decreased from 7 to 9. Namely, the harvest efficiency peaked at the nature pH value without any pH adjustment, valued at 98.73% after fitting (table 3). Thus, the highest harvest efficiency achieved when the medium pH closed to neutral condition, which is applicable since an adjustment of pH cost a lot. Therefore, 40 mg g^−1^ Al^3+^ and 60 mg CTAB/g dry biomass without pH adjustment was the optimal composite chemicals for coagulation, which can obtain a maximum harvest efficiency of 98.73%, higher than most studies using flotation with similar chemicals as listed in table 4.

**Table 3.** Fitting parameters of flotation recovery data of *Chlorella* sp. XJ-445 with the presence of 40 mg Al^3+^ and 60 mg CTAB/g dry weight biomass using equation (3.1).

pH value	*Y*_max_ (%)	*b* (min^−1^)	*R*^2^
5	21.39	0.275	0.8447
6	35.76	1.852	0.9570
6.77	98.73	0.431	0.9806
7	88.80	0.613	0.9901
8	63.83	0.761	0.9887
9	70.73	0.245	0.9033

**Table 4.** The comparison of results from algae flotation.

algae species	coagulant	removal (%)	zeta potential (mV)	references
*Chlorella vulgaris*	aluminium sulphate	94.8	—	[33]
*Scenedesmus quadricauda*	CTAB	90	−20	[34]
*Scenedesmus quadricauda*	CTAB	85	—	[35]
*Tetraselmis* sp.	aluminium sulphate	85.6	—	[36]
*Chlorella zofingiensis*	aluminium sulphate	91.5	−20.6	[37]
*Chlorella vulgaris*	CTAB	>93.7	−30.3	[30]
*Chlorella* sp.	CTAB	99.6	—	[11]
*Tetraselmis* sp.	CTAB	84.7	—	[11]
*Chlorella* sp.	CTAB	92	—	[32]
*Chlorella* sp.	CTAB + aluminium sulphate	98.3	−22.13	this study

### 3.3. Characterization of the algal cells

**Figure 5.**
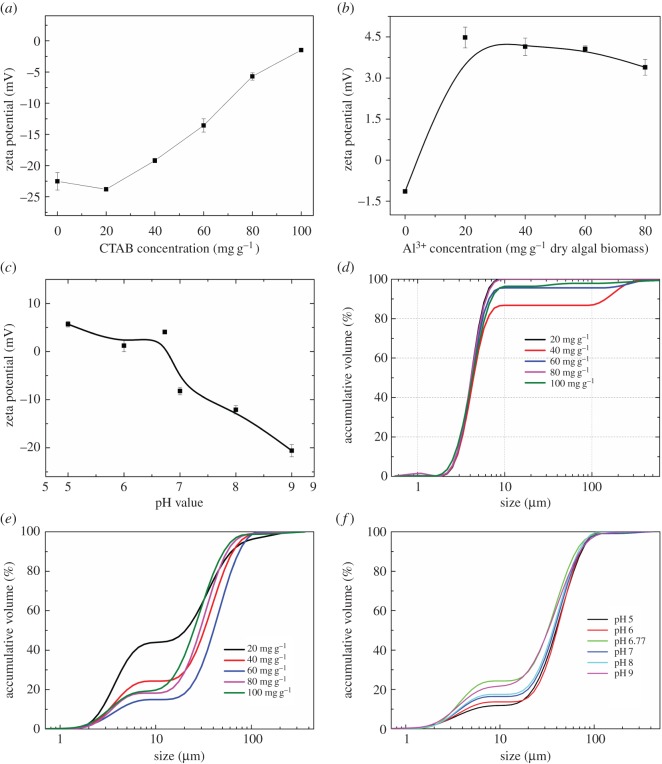
Zeta potential under different CTAB concentrations (*a*) and different Al^3+^ concentrations with 60 mg CTAG/g dry biomass (*b*) as well as different pH values (*c*); particle size of algal cells under different CTAB concentrations (*d*) and different Al^3+^ concentrations with the presence of 60 mg CTAB/g dry biomass (*e*) as well as different pH values (*f*).

To investigate the mechanism for the composite chemicals enhancing the flotation recovery efficiency, experiments for characterization of algal cells in terms of zeta potential, floc size and degree of hydrophobicity were conducted. Figure 5 presents the zeta potential and floc size of *Chlorella* sp. XJ-445 under different conditions. As shown, without any chemical addition, a zeta potential of −22.13 mV was observed at nature medium for *Chlorella* sp. XJ-445, which was among the values reported in the previous studies for *Chlorella* (table 4). With the increasing concentration of collector CTAB, net negative zeta potential decreased, and approached zero with a concentration of 100 mg g^−1^ dry cell weight CTAB (figure 5*a*). The downward trend of surface charge of microalgae cells with increasing CTAB concentration indicated that charge neutralization occurred. In addition, with the combination reaction of Al^3+^, zeta potential of algal flocs tended to be positive (figure 5*b*). 20 mg g^−1^ Al^3+^ addition exhibited a zeta potential of 4.5 mV; however, further increase in Al^3+^ concentration did not show any significant change. When adjusting the pH value to 5, the net positive zeta potential increased (figure 5*c*). In addition, with increasing pH value, zeta potential of algal flocs decreased and tended to be negative. CTAB is a cationic collector which neutralizes the negative charges of algal cells, and Al^3+^ further attracted the negatively charged cells, causing them to become destabilized and hence to coagulate, corresponding to figure 5*d*,*e*. Accumulative volume of particle size showed that most of the algal flocs (over 80%) were below 10 µm (figure 5*d*) with CTAB used alone. It was suggested that the use of CTAB for a microalgae flocculation is limited [7], so that the flotation efficiency was relatively lower and adding a coagulant was necessary and of significance.

As expected, the algal floc size sharply increased after Al^3+^ addition and showed an accumulative volume of over 80% above 10 µm, and increased with the increasing Al^3+^ (figure 5*e*), which was consistent with the previous studies [31,38]. The algal cells were supposed to be larger without DOM, which neutralized some coagulants for its negative charge [28,29]. However, when using flotation, a concentration of 40 mg g^−1^ Al^3+^ showed the best performance with medium floc size and medium iron strength, which favoured flotation. Moreover, even adjusting the pH value, an initial pH without pH adjustment showed the highest floc size and achieved the highest recovery efficiency (figure 5*f*).

**Figure 6.**
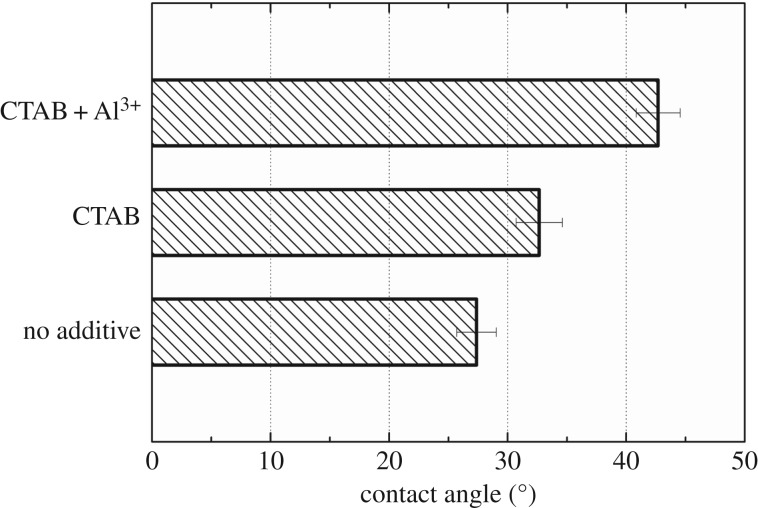
Contact angle changes of algal sheet under different conditions.

As for the hydrophobicity, figure 6 shows the contact angles of algal cells under different chemicals addition. The contact angle ranking from high to low was algal cells with combined use of 60 mg CTAB and 40 mg Al^3+^/g dry biomass, the addition of 60 mg CTAB/g dry weight biomass and algal cells without any chemicals addition. Previous study by Garg *et al*. has proved that algal hydrophobicity can be improved by using a cationic collector [11]. Coward *et al*. also reported that most of CTAB in the suspension was adsorbed onto the cells and modified the surface of algal cells [12]. Nevertheless, it cannot be neglected that the cationic surfactant is a bubble surface modifier which also enhances the hydrophobicity of the bubbles, and further enhancing the hydrophobic interaction between the bubble and algal cells largely favours the flotation [29]. Moreover, this study suggested the combined use of collector and coagulant can further enhance the degree of hydrophobicity of algal cells. Therefore, it was the higher floc size of algal cells and the higher hydrophobicity that enhanced the flotation efficiency when combining a chemical addition of CTAB and Al^3+^.

### 3.4. DLVO modelling

**Figure 7.**
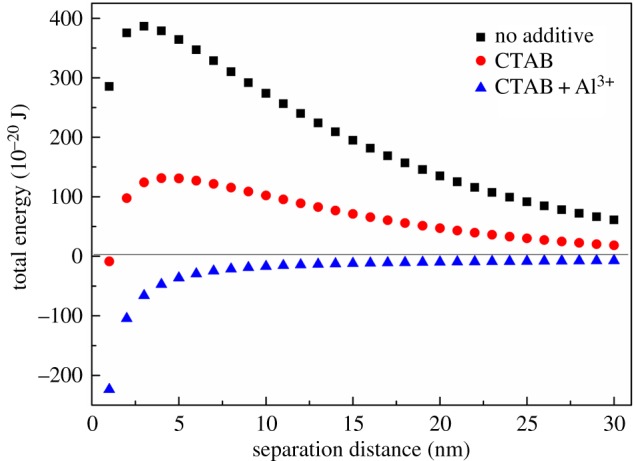
The total interaction energy of *Chlorella* sp. XJ-445 cells as a function of separation distance under different conditions.

Based on the DLVO theory, the electrostatic double layer surrounding the charged cells is related to the ionic concentration of the bulk solution as shown in equation (2.5). Owing to the fact that interactions between cells are primarily responsible for the coagulation behaviour, it was necessary to calculate the total interaction energy, which helps to predict whether the interaction between cells is attractive (*G*_TOT_ negative) or repulsive (*G*_TOT_ positive) as a function of separation distance as depicted in figure 7. The energy barrier of the microalgae suspension was reduced from 364 × 10^−20^ J to 131 × 10^−20^ J by only adding 60 mg g^−1^ dry cell weight biomass CTAB at the separation distance of 5 nm. When combined with 40 mg g^−1^ Al^3+^, the total energy further decreased to −36.3 × 10^−20^ J. This can be largely attributed to the sharply decreasing electrostatic repulsive forces between cells resulting from the decrease in the zeta potential as presented figure 5*b*. Thus, according to the DLVO model, the coagulant efficiency of *Chlorella* sp. XJ-445 was expected to increase from solely adding CTAB to combined adding CTAB and Al^3+^. Thus, the experimental results were in agreement with the model as shown in figure 5*d*–*f*. Therefore, the DLVO theory can be used to interpret the coagulation process using combined CTAB and Al^3+^.

### 3.5. Lipid content and fatty acid composition

The lipid content and mole percentage of prevalent of FAMEs isolated from C*hlorella* sp. XJ-445 for all the treatments were summarized in table 5. The lipid content showed no significant differences between adding 60 mg g^−1^ CTAB and combined 60 mg g^−1^ CTAB and 40 mg g^−1^ Al^3+^ (*p* > 0.05). However, the fatty acid composition significantly changed by chemical addition (table 5). When the algal cells were pretreated with single CTAB, significantly higher yields of the saturated fatty acid (SFA) and lower polyunsaturated fatty acid (PUFA) were recovered (*p* < 0.05). These observations were consistent with the previous study by Coward *et al*. [12]. In addition, combined addition of CTAB and Al^3+^ showed the similar pattern, however, greater degree in changing. The significantly higher content of C16 : 0 and C18 : 0 after the addition of Al^3+^ contributed to the total SFA, implying that the significant degree of change was largely attributed to the presence of Al^3+^. Rwehumbiza *et al*. [39] also reported that the residual aluminium affects composition of FAMEs of microalgae, which favours the biodiesel quality [39]. The higher osmotic pressure around the algal cells because of chemical addition could decrease the membrane permeability and fluidity, which would potentially avoid excessive ions into cells and cause changes in fatty acid composition, eventually. Though a decrease was found in monounsaturated fatty acid (MUFA), the total amount of SFA and MUFA from the combination of CTAB and Al^3+^ significantly increased (*p* < 0.05). In terms of biodiesel quality, higher proportions of MUFA and SFA and relatively lower content of PUFA are desirable as they increase cetane number, and also improve the oxidative stability, two of the most important parameters representing the diesel property [39]. Thus, flotation with reagents addition resulted in more predominance of saturated and monounsaturated fatty acids within the fatty acid profile, which provides many favourable features for biodiesel production.

**Table 5.** The influence of chemicals addition on the lipid content and fatty acid composition of *Chlorella* sp. XJ-445.

	no additive	60 mg g^−1^ CTAB	40 mg g^−1^ Al^3+^ + 60 mg g^−1^ CTAB
lipid content (mg g^−1^)	298.3 ± 3.8	287.5 ± 5.8	315.6 ± 19.6
lipid profile (mole percentage, %)			
14 : 0	4.84 ± 0.12	11.01 ± 0.48	4.91 ± 0.15
16 : 0	36.67 ± 1.53	34.67 ± 2.08	46.47 ± 3.35
16 : 1	6.87 ± 0.07	12.60 ± 0.53	1.65 ± 0.22
18 : 0	—	—	8.64 ± 0.18
18 : 1	23.24 ± 0.67	18.21 ± 0.36	17.79 ± 0.73
18 : 2	7.40 ± 0.62	10.88 ± 0.14	5.33 ± 0.20
18 : 3	13.52 ± 0.56	7.62 ± 0.07	6.19 ± 0.51
18 : 4	2.51 ± 0.11	1.33 ± 0.05	2.07 ± 0.81
others	4.94 ± 0.09	3.68 ± 1.37	6.96 ± 1.81
SFA	41.56 ± 1.59	45.68 ± 2.24	61.97 ± 1.60
MUFA	30.11 ± 0.69	30.81 ± 0.73	20.67 ± 2.62
PUFA	23.82 ± 0.54	19.83 ± 0.15	14.73 ± 2.52

## 4. Conclusion

Microalga with low surface hydrophobicity and small size as well as hydrophilic DOM in the medium are difficult to harvest by flotation separation. A pretreatment by mixing algal cells with Al^3+^ as coagulant and CTAB as collector was employed for algal surface modification. The floc size and degree of hydrophobicity of algal cells were largely enhanced and a maximum harvesting efficiency of 98.73% was achieved by the sequential dispersed air flotation without any pH adjustment. A better fatty acid composition in relation to biodiesel was further found. It was concluded that a coagulation-assisted flotation can lead to effective separation of microalgae from the dilute medium.

## Data accessibility

All of the data in this investigation have been reported in the paper and are freely available.
